# Quantitative Sensory Testing to Predict Postoperative Pain

**DOI:** 10.1007/s11916-020-00920-5

**Published:** 2021-01-14

**Authors:** Matthias Braun, Corina Bello, Thomas Riva, Christian Hönemann, Dietrich Doll, Richard D. Urman, Markus M. Luedi

**Affiliations:** 1 Department of Anaesthesiology and Pain Medicine, Inselspital, Bern University Hospital, University of Bern, Freiburgstrasse, 3010 Bern, Switzerland; 2 Department of Anaesthesiology, Catholic Clinics Oldenburger Münsterland gGmbH, St. Marienhospital Vechta, Teaching Hospital of the Medizinische Hochschule Hannover, Vechta, Germany; 3 Department of Surgery, Catholic Clinics Oldenburger Münsterland gGmbH, St. Marienhospital Vechta, Teaching Hospital of the Medizinische Hochschule Hannover, Vechta, Germany; 4 Department of Anesthesiology, Perioperative and Pain Medicine, Brigham and Women’s Hospital, Harvard Medical School, 75 Francis Street, Boston, MA 02115 USA

**Keywords:** Quantitative sensory testing, Acute postoperative pain, Persistent postoperative pain

## Abstract

**Purpose of Review:**

We review the relevance of quantitative sensory testing (QST) in light of acute and chronic postoperative pain and associated challenges.

**Recent Findings:**

Predicting the occurrence of acute and chronic postoperative pain with QST can help identify patients at risk and allows proactive preventive management. Generally, central QST testing, such as temporal summation of pain (TSP) and conditioned pain modulation (CPM), appear to be the most promising modalities for reliable prediction of postoperative pain by QST. Overall, QST testing has the best predictive value in patients undergoing orthopedic procedures.

**Summary:**

Current evidence underlines the potential of preoperative QST to predict postoperative pain in patients undergoing elective surgery. Implementing QST in routine preoperative screening can help advancing traditional pain therapy toward personalized perioperative pain medicine.

## Background

Pain continues to be a major problem in the perioperative period and after discharge, posing challenges to both patients and their treating physicians. More than 30% of patients have at least moderate postoperative pain, and a large proportion of these patients do not receive adequate pain management [1, 2].

Acute postoperative pain represents a psychological and physical burden for patients [3, 4]. It alters wound healing [5] and may further increase the frequency of cardiopulmonary and thromboembolic events [6, 7] as well as gastrointestinal and renal complications [8].

Persistent acute pain leads to prolonged recovery, to delayed discharge from the hospital, and to higher re-admission rates, which can significantly increase the costs of treatment [8–10].

Furthermore, acute postoperative pain is also related to a significantly elevated risk of developing chronic pain, seen in 10–50% of the individuals [3]. Along with up to 100 million adults reporting chronic or acute pain solely in the USA, there has been a massive increase in the use of opioids [11, 12] with many negative consequences.

In the USA, legal and illegal opiate consumption has become a major problem in recent years. According to the Centers for Disease Control and Prevention, almost 450,000 people died from opiate overdoses between 1998 and 2018, 33,000 of them in 2015 alone [11]. Every year, there are around 750,000 opioid-related visits to US emergency departments [11]. In particular, overdoses of highly potent opiates such as fentanyl have increased sharply [11]. This phenomenon primarily affects the USA, and to a lesser extent Australia and Canada.

European countries seem to be less affected, although in Germany the number of patients being treated with opioids has increased [13–15]. The overall economic burden has been estimated at 78.8 billion USD in the USA for the year 2013 [16]. The societal burden caused by opioids is higher than that of diabetes, cancer, and heart disease combined [11]. The inappropriate use of opiates and first-time exposure to opiates due to severe postoperative pain are risk factors for developing chronic opioid abuse [11, 12, 14, 17]. A multi-modal solution to this problem, embracing prevention, treatment, and rehabilitation [12], will undoubtedly be needed in the future.

Identifying risk groups in the patient population is a key step toward provision of individualized pain therapy [18] and may help reduce long-term opioid use. This pain experience may range from acute postoperative pain to an increased possibility of developing persistent postoperative pain (PPSP).

The sensation of pain is characterized by a large interindividual range. The same stimuli may be recognized as unpleasant in some individuals but not in others. Multiple factors influence the perception of pain, such as genetic determinants, neurological diseases affecting the central or peripheral nervous system, inflammatory modulation of tissue, and the presence of inflammatory mediators, as well as experience of pain, i.e., peripheral sensitization of pain possibly leading to central modulation of pain pathways [2, 19–25]. Also, psychological factors such as resilience, depression, pain catastrophizing, mood, positive or negative emotions, and stress affect the sensation of pain [26–29].

The current literature addressing pain is ambiguous, and there is still a gap in knowledge of the mechanisms behind perioperative pain. However, implementing quantitative sensory testing (QST) in the preoperative setting may help detect potential pain-related problems at an early stage, allowing treatment to be provided sooner. The establishment of multimodal individualized options could help reduce the prevalence of PPSP in the acute and chronic setting and fight subsequent pain-relieving drug abuse. Therefore, we aimed to provide an overview of different QST modalities and the evidence for their use in the perioperative setting.

### Quantitative Sensory Testing

QST involves procedures testing perception, pain thresholds, and pain tolerance thresholds for different stimuli. These are based on standardized pressure, vibration, thermal, or electrical impulses. Classically used for the diagnosis of specific nerve fiber function or dysfunction, the detection of neurological diseases and neuropathic pain, these procedures have the potential to identify patients at risk of postoperative pain [21, 30–33].

Stimuli can be measured at the patient’s detection limit, at the pain threshold, above the pain threshold, and if needed at the pain tolerance level. Classically, for neuropathology diagnostics, the detection threshold rather than the limit for noxious stimuli is used, whereas some data for perioperative QST testing seem to show better results with suprathreshold pain stimuli [2, 21]. Different fibers can be tested by selected QST modalities. The cold detection threshold represents the function of Aδ fibers; the heat detection threshold represents the function of C fibers; the heat pain threshold is mainly a function of nociceptive C fibers, and mechanical detection and vibration is an Aβ fiber function [19, 34]. The common thermal QST tests are referred to as the warm detection threshold (WDT), cold detection threshold (CDT), heat pain threshold (HPT), cold pain threshold (CPT), or suprathreshold heat pain intensity (STHPI). Mechanical testing is often measured with Frey filaments, and pain threshold with blunt needles or cuffs. For allodynia, soft materials such as cotton beads or brushes are used, while tuning forks are used for vibration testing. Commonly applied mechanical QST tests are pressure pain threshold (PPT), suprathreshold pressure pain intensity (STPPI), and pressure pain tolerance (PPTol). Electrical testing includes electrical detection threshold (EDT), electrical pain threshold (EPT), and electrical pain tolerance (EPTol) [2, 19, 25]. For the quantification of pain intensity, the Numerical Rating Scale (NRS) and Visual Analogue Scale (VAS) are regularly used [2].

In 2006, the German Research Network of Neuropathic Pain (GRNNP) introduced the first protocol for standardized QST testing of neuropathic pain patients. The protocol includes seven tests assessing mechanical and thermal threshold, as well as pain detection in different manners, using hot and cold stimuli, pressure, vibration, and pain summation to pinprick, for a total of 13 parameters [35]. In 2016, the GRNNP protocol was used in 10 European centers and showed highly comparable data with low heterogeneity [36].

This standardized protocol has been customized for patients with neuropathic pain. However, high-quality equipment is required and testing is time consuming, so that a complete bilateral examination of hand, feet, and face takes around 3 h, or half an hour per test [35, 36]. Unfortunately, for these reasons, a standardized protocol for the large-scale use of pre- and perioperative QST assessment is lacking, which limits its usefulness in daily clinical practice and its reproducibility for further clinical trials.

Apart from different pain modalities, central pain processing plays an important role and can be predicted by more dynamic testing such as temporal summation of pain (TSP) [37] and conditioned pain modulation (CPM) [2]. These procedures seem to find their way into clinical studies more frequently [2, 20–25].

With TSP, the patient repeatedly receives equally painful stimuli, which leads to an increased perception of pain. These stimuli can be combined in various ways, using pressure, heat, or electric current. It has been shown that this approach allows conclusions about the perception of pain in ascending neural pathways, and about the processing of pain in descending neural pathways [38]. TSP shows promising results in both healthy and chronic pain patients [39]. The concept of CPM is based on the assumption of testing the diffuse noxious inhibitory control (DNIC) system [40]. The DNIC system inhibits a painful stimulus as a second painful stimulus is applied. Although there are large interindividual differences, CPM seems to provide reliable information for chronic pain and pain management [40, 41]. Such dynamic tests allow more individualized testing of pain sensation and processing. Unlike other sensory tests, they are more independent of factors such as position or modality of the applied stimulus [42].

Many other factors influence postoperative pain and pain perception. Preoperative pain tolerance and preoperative pain experience seem to correlate with postoperative pain intensity and the use of postoperative analgesics [20]. In a systematic literature review, Von Helmond described a significant association of QST (TPS, CPM, and PPT) and PPSP in populations suffering from considerable preoperative pain in 10 out of 14 studies [25•].

Other data have shown preexisting preoperative pain, or inflammatory modulation of the tissue and inflammatory agents, type of surgery, psychological distress (e.g., anxiety), and pain catastrophizing, age, opioid use, and post-traumatic stress disorder as risk factors for both acute and persistent postoperative pain [2, 20, 21, 24, 25, 43–46]. Pain catastrophizing describes a state of perceiving pain as a constant overpowering danger, leading to compulsive avoidance of (potential) pain, for example through inactivity. This may also intensify pain awareness, which in turn increases avoidance behavior [43, 47]. This can lead to a vicious circle. To assess psychological factors, pain catastrophizing scales—or for shorter-term evaluation the ‘Daily Pain Catastrophizing Scale’—are used more and more often as predictive instruments, also in combination with QST [31, 48–52].

In the past two decades, extensive research has been conducted on perioperative QST, and some systematic reviews can be found [2, 7, 20–25]. The literature, including these reviews, covers a broad range, including response to analgesic treatment of postoperative pain [23] and analgesic consumption in acute and persistent postoperative pain [20, 22].

Studies involving preoperative QST investigate different types of surgical settings. They cover orthopedic procedures, mostly open, such as knee and hip replacement, but also arthroscopic knee and shoulder surgery, gynecological procedures, often including cesarean section, hysterectomy/myomectomy, tubal ligation, or other laparoscopic interventions. Abdominal surgery such as open or laparoscopic hernia repair, open gastrointestinal surgery, and other procedures such as carpal tunnel release or lumbar discectomy and limb amputation are also included [2, 20, 21, 23–25, 53].

The best correlation between QST and postoperative pain seems to be found in orthopedic surgery. Van Helmond found a correlation in seven out of ten studies with orthopedic interventions (including six knee replacements) [25•], and Petersen found reliable preoperative predictors primarily for orthopedic surgery, followed by abdominal and gynecological procedures [24•]. Yet the influence of the procedure itself, the specific QST modality, or a combination thereof remains unclear [24•].

Different types of surgery lead to destruction of various structures in the body. Whether different QST modalities such as cutaneous stimuli or pressure-induced activation in deeper tissue layers have specific predictive power for specific interventions, leading to better correlation, as for example in joint surgery, is still unclear and requires further investigation [24•]. Sangesland et al. postulated that not all QSTs are equally sensitive to different organ systems. They found a positive correlation between PPT and postoperative pain in the acute and chronic setting after musculoskeletal surgery, but not after visceral surgery [2]. However, in a recent study with 128 patients, Luedi et al. showed a significant correlation between PPT and pain up to 1 month postoperatively in a short-stay anorectal surgery cohort [53•]. Also, in contrast to PPT, CPM showed an inverse correlation [53•]. Further research should investigate whether mainly ambulant or short-stay procedures were included, in order to gain clarity.

In 2010, a systematic review showed better predictive strength of QST overall for persistent postsurgical pain compared with any other single parameter, such as gender, age or psychological factors. In 14 studies, 4–54% of the variance in postoperative pain could be predicted with preoperative exposure to painful stimuli [21]. As described at that time, of the QST methods and protocols used, the most promising results were found with the application of STHPI and electrical stimulation [21].

There are still more recent contradictory data. Grosen et al. reviewed 14 articles stating the validity of heat and the electrical pain threshold (PPT and STHPI) in surgical patients to predict analgesic effects, but overall, the data were insufficient to recommend a specific modality of QST [23]. Sangesland postulated that threshold detection for electrical and thermal pain poorly predicted postoperative pain intensity [2], but the authors found better correlations with suprathreshold (heat) stimuli [2].

Latest research puts the focus on PPSP, which appears to last more than 3 months after surgery [24, 25]. Many factors, such as the duration of acute postoperative pain [54] or immediate postoperative intensity of the pain [42], might play a role in the development of PPSP. QST might help to detect potential strong and prolonged pain experience in advance, as the association of postoperative pain and chronic pain is well established [55]. In 2020, Van Helmond showed a correlation between QST and PPSP in 14 of 24 studies reviewed (58%), once again with orthopedic procedures having the most powerful correlation. Although there is a correlation not only between QST and acute pain but also with PPSP, this correlation seems to be higher than at a later stage [25•].

Petersen et al.’s recent review of 25 surgical and 11 pharmacological studies, also focusing on chronic postoperative pain and pharmacological treatment in chronic pain patients, found a correlation with QST measurements in 17 and 11 studies, respectively. The best correlation could be shown with dynamic testing such as TSP (50%) and CPM (44%) [24•].

In general, the most promising prediction of postoperative pain can be achieved with dynamic central testing, such as TSP and CPM [31, 32, 56–58]. Dynamic testing is becoming more common [2, 24•]. TSP is the only QST modality with fewer insignificant than significant chronic pain associations [25•]. Interestingly, there seems to be an association of PPSP-predictive CPM and TSP findings preoperatively in patients suffering from pain. Either preoperative pain could lead to central modulation of pain perception [25•] or—as chronic pain patients often show hyperalgesia [24•, 59]—patients with persistent chronic pain may simply be more receptive to pain in advance, making them vulnerable to developing PPSP [24•]. Further research is needed to prevent bias from a lack of understanding of the underlying mechanisms influencing postoperative pain.

### Limitations

A major problem of QST research is the heterogeneity of the data and the complexity of the studies. Several QST protocols combine different test settings and modalities. Timing of the preoperative QST, for example, varied widely or was not reported [2], although the timing may be essential [24, 60]. The testing itself does not show high consistency, but the results may change over time in an individual. Good reproducibility of QST in healthy patients may last just days or weeks [58]. Not only duration of testing but also location may have an impact. For example, in an orthopedic surgery, the application of the QST may show different results on the surgical and contralateral sides. Many studies include a high rate of bias. It has been suggested that study protocols should be harmonized for a better understanding of QST, or making them shorter and easier for clinical use [34].

Most of the included studies had at least moderate or even high bias. This may lead to an overestimation of the correlation in the studies with a higher risk of bias. Ninety-two percent of the studies evaluated by van Helmond have a moderate or high risk of bias [25•]; only Petersen et al. classified studies as low to moderate risk [24•]. Sangesland et al. classified only seven of 30 studies as low bias (4 of them showed positive results for QST) [2]. In addition, studies with a high risk of bias often have suboptimal predictive value [24•].

In short, low risk of bias and strong correlation between QST and postoperative pain were found for CPM in knee arthroplasty and gastrointestinal surgery, PPT in total knee replacement, whereas moderate correlation could be reported for PPT and carpal tunnel procedures as well as WDT and surgical treatment of lumbar disc herniation [24•]. Furthermore, a large number of studies did not sufficiently show absence of predictive value of PPSP and QST [25•]. In two recent systematic reviews, not a single preoperative QST parameter was found to be consistent. The strengths of models used in the studies showed large variation in predictive ability [2, 24]. Table 1 provides an overview of positive association of QST and postoperative pain as defined in systematic reviews.

**Table 1:** Positive association of QST and postoperative pain defined in systematic reviews

Systematic review	*n*=	QST testing used	Positive association between QST and postoperative pain
2016 Sangesland	30 (2738)	**TST** (WDT HPT, STHPI, CDT, CPT, STCPI, CPTol **EST** (EDT, EPT, EPTol) **MST** (PPT, STPPI, PPTol) **TSP** **CPM** **CA**	**TST 10/34** (WDT 0/3, HPT 2/13, STHPI 7/12, CDT 0/1, CPT 0/3, STCPI 0/1, CPTol 1/1) **EST 4/13** (EDT 1/4, EPT 3/7, EPTol 0/2) **MST 4/18** (PPT 2/11, STPPI 0/2, PPTol 2/5) **TSP 3/5** **CPM 3/7** **CA 1/1**
2020 Peterson	25	***TST*** (WDT, HPT, CDT, CPT, STHCS) **EST** (EDT, EPT, EPTol) **MST** (MDT, MPT, PPT, PTT, CIPDT, CIPTT, DMA) **TSP** **CPM**	***TST 5/11*** (WDT 1/3, HPT 1/9, CDT 0/2, CPT 0/4, STHCS 1/3) **EST 2/4** (EDT 0/1, EPT1/2, EPTol 0/1) **MST 5/28** (MDT 0/2, MPT 0/4, PPT 3/11, PTT 0/2, CIPDT 1/3, CIPTT 0/3, DMA 1/3) **TSP 5/9** **CPM 4/12**
2020 Van Helmond	24	***MST*** *(VT, TP, PPTT, PPT, PDT, MPT, MDT)* ***EST*** *(EPTT, EPT, EDT****)*** ***TST*** *(WDT, STHPI, STCPI, HPT, CPT, CDT)* ***TSP*** ***CPM***	***TST 3/25*** *(WDT 2/4, STHPI 0/4, STCPI 0/2, HPT 1/8, CPT 0/4, CDT 0/3)* ***EST 2/6*** *(EPTT 0/2, EPT 1/2, EDT 1/2)* ***MST 5/26*** *(VT 0/1, TP 2/2, PPTT 0/4, PPT 3/14,* *PDT 0/1, MPT 0/3, MDT 0/1)* ***TSP 5****/****7*** ***CPM 3/7***
2013 Grosen	14	***TST*** *(HPT, TSP, CPT* *STCPI, HST)* ***EST*** *(EPT, EPTol)* ***MST*** *(PPT, PuPT,* *Tactile allodynia)*	***TST 4/7*** *(HPT 3/4, STCPI 1/3)* ***EST 2/10*** *(EPT 2/5, EST 0/3, EPTol 0/2)* ***MST 1/5*** *(PPT 1/3, PuPT 0/2)*
2010 Werner	14	***TST*** *(HSPT, HTS, HPT, CPPT, WDT, CDT, CPT)* ***MST*** *(PPT, MPT, MSTP)* **TSP** ***CPM***	***TST 12/31****(HSPT 8/13, HTS 0/1, HPT 3/10, CPPT 1/1, WDT 0/4, CDT 0/1, CPT 0/1)* ***MST/2/5*** *(PPT 1/1, MPT 0/3, MSTP 1/1)* ***EST 6/11****(EDT 1/4, EPT 4/5, EPTol 0/1, ESTP 1/1)* **TSP 1/1** ***CPM 1/1***

*TST* thermal sensory testing, *WDT* warm detection threshold, *HPT* heat pain threshold, *STHPI* suprathreshold heat pain intensity, *CDT* cold detection threshold, *CPT* cold pain threshold, *STCPI* suprathreshold cold pain intensity, *CPTol* cold pain tolerance, *EST* electrical sensory testing, *EDT* electrical detection threshold, *EPT* electrical pain threshold, *EPTol* electrical pain tolerance, *EPT* electrical pain threshold, *ESTP* electrical suprathreshold pain perception, *MST* mechanical sensory testing, *MSPT* mechanical suprathreshold pain, *PPT* pressure pain threshold, *STPPI* suprathreshold pressure pain intensity, *PPTol* pressure pain tolerance, *TSP* temporal summation of pain, *CPM* conditioned pain modulation, *CA* cutaneous allodynia, *STHCS* suprathreshold heat and cold stimuli, *MDT* mechanical detection threshold, *MPT* mechanical pain threshold, *PTT* pressure tolerance threshold, *CIPDT* cuff induced pain detection threshold, *CIPTT* cuff induced pressure tolerance threshold, dynamic mechanical allodynia, *PDT* pressure detection threshold, *PPTT* pressure pain tolerance threshold, *PuPT* punctuate pain threshold, *TP* tonic pain, *VT* vibration threshold

QST remains dependent on the current state of the patient. Sufficient mental ability to perform all tests, as well as factors such as motivation and compliance, plays a key role [19].

### Implementation in Clinical Practice

Although there is an association between QST and acute PPSP, most experts cannot recommend routine use for regularly scheduled surgeries. Due to the fact that most QST is time consuming and demands considerable resources, further studies with multivariate analyses of different central QST modalities are needed [2, 24, 25]. These should be combined with factors not associated with QST. A preselection of the patients receiving QST might improve predictive power.

## Conclusion

In summary, while extensive research has been conducted in the field of preoperative QST testing and its relationship to acute postoperative pain and PPSP, central QST methods such as TPS and CPM show the most promising predictive potential. Future high-quality studies combining central QST procedures, such as TPS and CPM, can help advancing traditional pain therapy towards personalized perioperative pain medicine. These projects should include study designs which combine non-QST-associated parameters such as anxiety, pre-existing preoperative pain, or pain catastrophizing and QST need to be standardized in order to reduce potential bias. Comprehensive preoperative QST testing of patients undergoing elective surgery might become a milestone in personalized perioperative pain medicine, with the potential to reduce the increasing burden of pain and improve quality of life.

## Data Availability

Not applicable.

## Abbreviations

CDT: Cold detection threshold
CPM: Conditioned pain modulation
CPT: Cold pain threshold
DNIC: Diffuse noxious inhibitory control system
EDT: Electrical detection threshold
EPT: Electrical pain threshold
EPTol: Electrical pain tolerance
HPT: Heat pain threshold
MST: Mechanical sensory testing
NRS: Numerical Rating Scale
PPSP: Persistent postoperative pain
PPT: Pressure pain threshold
PPTol: Pressure pain tolerance
QST: Quantitative sensory testing
STHPI: Suprathreshold heat pain intensity
STPPI: Suprathreshold pressure pain intensity
TSP: Temporal summation of pain
VAS: Visual Analogue Scale
WDT: Warm detection threshold
